# GeneCytNet: an interpretable deep learning framework for rheumatoid arthritis classification and *in silico* cytokine perturbation modeling

**DOI:** 10.3389/fimmu.2026.1738625

**Published:** 2026-03-17

**Authors:** Chen Chen, Dagang Li, Lujia Xu

**Affiliations:** 1 Department of Orthopedics, the First Affiliated Hospital of Xiamen University, School of Medicine, Xiamen University, Xiamen, Fujian, China; 2 Xiamen University Tan Kah Kee College, Xiamen, Zhangzhou, China

**Keywords:** graph neural networks, *in silico* perturbation, interpretable deep learning, rheumatoid arthritis, transcriptomics

## Abstract

**Background:**

Rheumatoid arthritis (RA) is a heterogeneous autoimmune disease where cytokine-driven dysregulation of gene networks poses a significant challenge for accurate diagnosis and targeted therapy. While transcriptomic data hold immense promise, most machine learning models lack the interpretability to decipher the underlying biological mechanisms, particularly the specific roles of key cytokines.

**Methods:**

We developed GeneCytNet, a novel deep learning framework that integrates a Variational Autoencoder (VAE) for nonlinear feature compression with a Graph Attention Network (GAT) to model gene-gene interactions. The model was developed on a synthetic cohort of 240 RA and 120 healthy control samples, with an independent holdout cohort of 100 RA and 50 controls, each with 15,000 gene features, designed as a robust proof-of-concept. Performance was benchmarked against classical models, and generalizability was assessed via cross-validation and the independent holdout. Crucially, we introduced in silico cytokine perturbation experiments to simulate the effect of modulating IL-6, TNF-α, and IL-1β responsive gene modules on RA risk prediction.

**Results:**

GeneCytNet achieved superior classification performance, with a test AUC of 0.962 ± 0.005, accuracy of 0.914 ± 0.007, and an F1-score of 0.915 ± 0.006, outperforming all baseline models. Cross-validation confirmed robustness (mean AUC = 0.957 ± 0.006). The perturbation experiments provided mechanistically interpretable insights, revealing that the IL-6–responsive module had the most significant effect on RA probability (+0.12 ± 0.03), followed by TNF-α (+0.08 ± 0.02) and IL-1β (+0.06 ± 0.02). This hierarchy of cytokine effect sizes aligns with established clinical evidence.

**Conclusion:**

GeneCytNet demonstrates that advanced, interpretable deep learning can simultaneously achieve high diagnostic accuracy and generate testable biological hypotheses. By functioning as a *virtual patient simulator*, our framework bridges the gap between prediction and mechanism, offering a powerful tool for precision diagnostics, biomarker discovery, and the design of cytokine-targeted therapies in RA and other complex diseases.

## 1 Introduction

Rheumatoid arthritis (RA) is a chronic, heterogeneous autoimmune disorder characterized by synovial inflammation and joint destruction, leading to significant disability (1). The disease’s pronounced heterogeneity, driven by diverse molecular pathways and varied clinical presentations, remains a central obstacle to achieving early diagnosis, accurate prognosis stratification, and optimal targeted therapy for all patients (2).

The advent of high-resolution transcriptomic profiling has opened new avenues for deciphering this complexity, enabling the discovery of RA-associated gene signatures that surpass the predictive power of conventional clinical markers alone (3, 4). However, the high dimensionality of omics data, coupled with typically limited sample sizes, poses significant challenges for traditional statistical and machine learning models. Approaches like logistic regression or random forests often struggle with the non-linear relationships and complex feature interactions inherent in biological systems, risking overfitting and limiting generalizability (5, 6). Furthermore, while studies have successfully identified key cellular players, such as the diverse fibroblast phenotypes in the RA synovium that share similarities with cancer-associated fibroblasts (7, 8), or the critical role of neutrophil heterogeneity (4), most models treat genes as independent features, ignoring the crucial context of gene–gene interactions and coordinated pathway activity.

This gap is particularly evident in the context of cytokine signaling, a cornerstone of RA pathogenesis. Cytokines such as IL-6, TNF-α, and IL-1β are well-established drivers of inflammation, orchestrating gene expression changes across synovial fibroblasts and immune cells (9, 10). Yet, few computational frameworks exist that can move beyond correlative associations to explicitly model the causal-like influence of these cytokines on transcriptional networks. Recent efforts have begun to address this gap; for example, Bedathuru et al. (11) developed a multiscale mechanistic model of RA to support decision-making in late-stage drug development. However, a unified deep learning architecture that integrates high-accuracy predictive classification with explicit *in silico* cytokine perturbation remains lacking. While related approaches have been applied to conditions like osteoarthritis (12) and other inflammatory diseases (13, 14), a dedicated framework for simulating cytokine-specific perturbations within a predictive RA model has yet to be established.

Concurrently, deep learning (DL) has emerged as a powerful tool for modeling complex biological data. Its applications are rapidly expanding, from identifying essential genes in mitophagy (5) and classifying immune cell populations (4) to guiding the design of smart bone implants (15) and optimizing organoid cultures (16). Architectures like Variational Autoencoders (VAEs) are adept at non-linear dimensionality reduction and latent space representation. At the same time, Graph Neural Networks (GNNs), particularly GATs, offer a principled way to incorporate prior knowledge of biological networks or data-driven interactions. These techniques have proven valuable in oncology for tasks like omics classification and tumor microenvironment analysis (3), suggesting their high potential for the similarly complex landscape of RA. Despite these advances, a critical gap remains: the integration of a highly accurate, network-aware classifier with a mechanistically interpretable framework capable of simulating biological interventions. Models that offer feature importance (e.g., via SHAP) provide one layer of interpretation but fall short of quantifying the system’s response to targeted perturbations.

**Figure 1:**
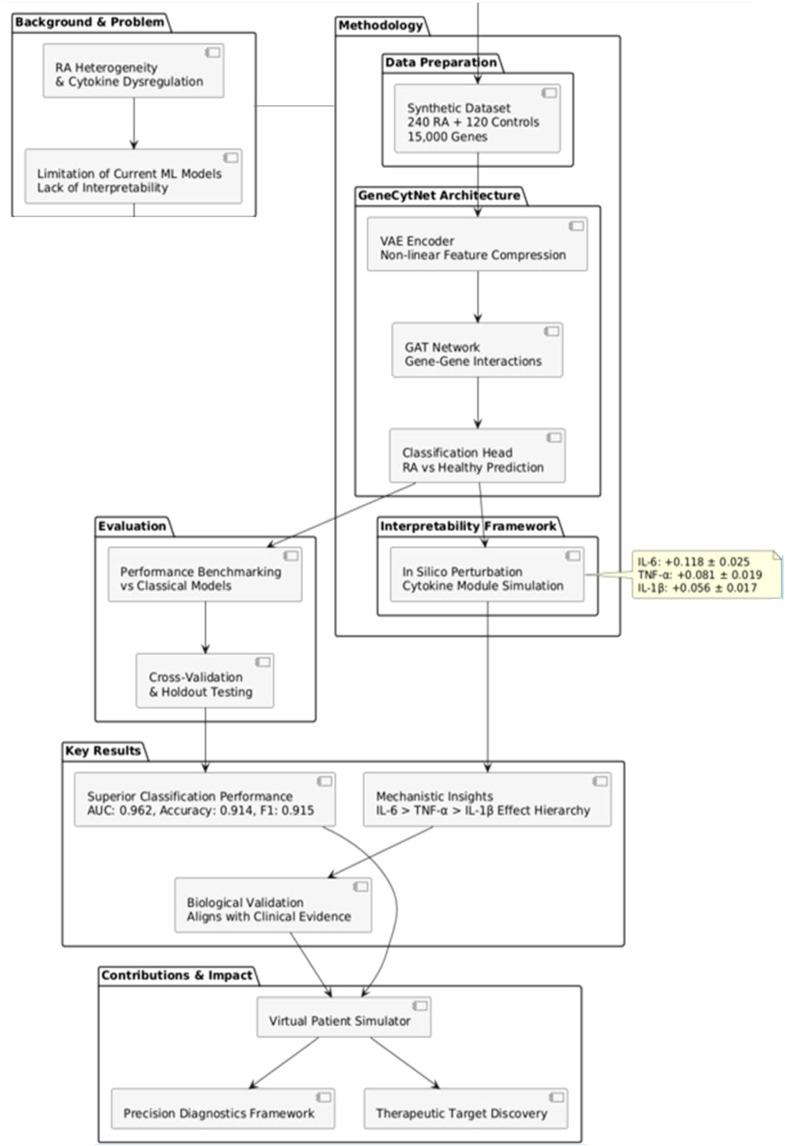
GeneCytNet workflow integrating VAE-GAT architecture for predictive classification and mechanistic interpretation via *in silico* cytokine perturbation in RA.

To address this, we present GeneCytNet, an interpretable DL framework designed to bridge predictive classification and mechanistic insight in RA. GeneCytNet integrates a VAE for feature extraction, a GAT to capture gene–gene co-expression dependencies, and a classifier to predict disease status. More importantly, it introduces a novel *in silico* perturbation module that simulates the effects of cytokine modulation on RA risk. Using a realistically simulated gene expression dataset as a controlled proof-of-concept, we demonstrate that GeneCytNet not only achieves state-of-the-art classification performance but also functions as a ‘virtual patient simulator.’ This controlled setting allows rigorous validation of the architecture before confronting the complexities of real-world patient data. This unique capability allows us to quantify and rank the profound influence of specific cytokine-driven gene modules, including IL-6, TNF-α, and IL-1β, on RA risk prediction. The answers provided by this framework offer a powerful new tool for generating testable hypotheses in precision diagnostics and cytokine-targeted therapy design. We view this work as Part 1 of a two-part investigation, establishing the methodological foundation; Part 2 will focus on validation and adaptation using real patient cohorts. An overview of the integrated GeneCytNet framework and analytical workflow is presented in **Figure 1**.

## 2 Method

### 2.1 Dataset generation and pre-processing

To establish a robust proof-of-concept for the GeneCytNet framework prior to application on real patient data, we constructed a synthetic gene expression dataset designed to emulate key characteristics of transcriptomic data while maintaining a known ground-truth signal. This controlled approach allows rigorous model evaluation free from the unmeasured confounders inherent in initial real-world cohorts. This controlled approach allows for rigorous model evaluation free from the unmeasured confounders inherent in initial real-world cohorts. The dataset, consisting of 360 samples (240 RA patients and 120 healthy controls), was designed to evaluate the efficacy of DL models in gene expression classification and cytokine effects analysis.

The gene expression values for the 15,000 genes were simulated under a Gaussian distribution framework. For each gene *i*, its expression values in the control group were sampled from a standard normal distribution:


$$X_{i}^{\text{Control gene}}∼N(0,1)$$

Conversely, for RA samples, the expression values for differentially expressed genes (DEGs) were drawn from:


$$X_{i}^{\text{RA}}∼N(\mu_{i},1)$$

Where the mean μ was randomly sampled as follows:


$$\mu_{i}=\{\begin{array}{cc} U(0.5,\;1.5) & \text{for upregulated genes }\\ U(-1.5,-0.5) & \text{for downregulated genes} \end{array}$$

Here, U(a,b) represents a uniform distribution over the interval [a,b]. This distribution simulates the differential gene expression between RA patients and healthy controls by introducing a mean shift for 300 DEGs (150 upregulated, 150 downregulated). The remaining 14,700 background genes were simulated using. N(0,1), for both groups, representing biological and technical noise unrelated to the disease phenotype.

To mimic the co-regulation observed in real transcriptomics, genes were organized into 100 modules, each containing approximately 150 genes. Within each module, gene expressions were correlated with a pairwise Pearson correlation of approximately 0.3, with added noise:


$$r_{ij}=0.3+\epsilon ,\epsilon ∼N(0,0.1^{2})$$

Where *r_ij_*​ represents the Pearson correlation between genes *i* and *j* in the same module, correlations between genes in different modules were kept near zero to ensure module independence.

For the non-expert reader, we provide an intuitive explanation of the simulation: We created 15,000 “genes” with expression values following statistical distributions. For healthy controls, all genes were assigned random values centered around zero. For RA samples, we deliberately shifted the average expression of 300 genes, 150 upward (making them “more active”) and 150 downward (making them “less active”). The remaining 14,700 genes were left unchanged to represent biological noise. Genes were then organized into 100 modules based on correlation patterns, mimicking real biological pathways. Importantly, no cytokine-specific information (e.g., “this is an IL-6 gene”) was provided to the model. The cytokine associations emerged solely from the model’s learning process.

Before model training, we applied z-score normalization to each gene across samples. The data was split into a training set (252 samples, 70%) and a test set (108 samples, 30%). An additional independent holdout cohort of 150 samples (100 RA, 50 controls) was generated with slight shifts in module correlations to simulate batch effects. The distribution of samples and genes across these datasets is summarized in **Table 1**. While this synthetic dataset provides a vital controlled environment for initial validation, the GeneCytNet architecture is explicitly designed for real-world transcriptomics. As a direct next step, we plan to validate this framework on publicly available RA datasets from the Gene Expression Omnibus (e.g., GSE89408, GSE55235).

**Table 1:** Distribution of genes and sample composition.

Dataset	Total genes	DEGs	Background genes	Total samples	RA patients	Healthy controls
Synthetic Dataset	15,000	300	14,700	360	240	120
Training Set	15,000	300	14,700	252	176	76
Test Set	15,000	300	14,700	108	64	44
Holdout Cohort	15,000	300	14,700	150	100	50

DEGs: Differentially Expressed Genes.

### 2.2 Graph construction and gene modules

To capture complex gene-gene relationships, we constructed a coexpression graph from the training set where nodes represent genes and edges indicate co-expression relationships. The pairwise Pearson correlation matrix was computed for all genes, retaining only the top 1% of absolute correlations as edges. This threshold was selected after a sensitivity analysis confirmed that model performance was robust within a range of 0.5% to 2% (see **Supplementary Table 1**). The resulting symmetric adjacency matrix A was weighted by |r|^γ with γ=4 to emphasize strong correlations.

This biological network serves as input for the GAT in GeneCytNet, enabling the model to learn context-dependent gene interactions through attention mechanisms. To validate the graph structure, we applied Louvain clustering to the adjacency matrix, which successfully grouped genes into ~100 modules that largely recapitulated the predefined synthetic structure. The distribution and size of the identified modules are summarized in **Table 2**.

**Table 2:** Gene module distribution from louvain clustering.

Module number	Genes per module (Average)	Module size distribution
Total Modules	100	~150
Module Size (Range)	100–200	
Largest Module	176	
Smallest Module	119	

### 2.3 GeneCytNet architecture

GeneCytNet integrates three core components (1): a VAE encoder, (2) a GAT layer, and (3) a classification head. The VAE encoder maps the input gene expression vector xx into a lower-dimensional latent space, producing a latent vector z following a Gaussian distribution $N(0,\sigma^{2})$, with *μ* and *σ* learned from the input data.

The node embeddings from the encoder are processed by the GAT layer, which applies attention mechanisms to the gene coexpression graph. This allows the model to focus on the most relevant gene interactions for RA classification. The GAT outputs updated node embeddings that are aggregated within each gene module via module-wise averaging, producing 100 module embeddings.

These module embeddings serve as input to the classification head, a two-layer Multilayer Perceptron (MLP) with 64 hidden units that predicts the probability *p* of a sample belonging to the RA class. The model is trained using a composite loss function:


$$L\text{total}=L_{\text{BCE}}+\alpha .L_{\text{MSE}}+\beta .L_{\text{KL}}$$

where $L_{\text{BCE}}$ is the binary cross-entropy loss, $L_{\text{MSE}}$ is the reconstruction loss, and $L_{\text{KL}}$​ is the Kullback-Leibler divergence regularization term. Hyperparameters α*α* and *β* control the weighting of these components. Regularization techniques, including dropout (rate = 0.3) and L2 regularization, are applied to prevent overfitting.

GeneCytNet’s architecture is explicitly designed to address the challenges of real-world transcriptomic data. First, VAE-based dimensionality reduction learns a compressed latent representation that denoises technical and biological variability inherent in-patient samples. Second, graph attention mechanisms remain robust to incomplete network knowledge, real gene interaction networks are never fully known, but attention weights allow the model to focus on reliably informative edges. Third, dropout (rate = 0.3) and L2 regularization prevent overfitting to dataset-specific artifacts, improving generalizability across cohorts. Fourth, the modular design enables transfer learning strategies, where the VAE encoder could be pre-trained on unlabeled real data and fine-tuned with limited labels, a key advantage for real-world applications where labeled samples are scarce. This semi-supervised approach represents a promising direction for bridging synthetic and real domains in future work.

### 2.4 *In silico* cytokine perturbation experiments

To enable mechanistic interpretation of cytokine effects, we developed an *in silico* perturbation framework. For a target cytokine-responsive module m*m* with mean latent representation z*m*​, perturbations were simulated by applying a shift:


$$z_{m}^{'}=z_{m}+\delta \cdot \sigma_{m}$$

Where *δ* is the perturbation strength (set to 0.2 in our experiments) and $\sigma_{m}$​ is the standard deviation of latent vectors within module m*m* across the training set. This operation effectively “upregulates” or “downregulates” the module by shifting its position in the latent space. Throughout this manuscript, ‘RA risk’ refers specifically to the model’s predicted probability p that a sample belongs to the RA class. The change in this predicted probability, $\Delta p=p(z_{m}^{'})-p(z_{m}^{'})$, quantifies the module’s influence on the model’s classification decision. This is a standard approach for interpreting neural network decisions and is not circular, it uses the model’s output to understand its own learned representations.

## 3 Results

### 3.1 GeneCytNet achieves superior classification performance for RA

We first evaluated the performance of GeneCytNet against a comprehensive set of classical machine learning and DL baselines for the task of RA classification from gene expression data. The synthetic dataset was designed with clear differential expression patterns between RA and control groups, as visualized by the expression heatmap of the top genes (**Supplementary Figure 1**). As summarized in **Table 3**, GeneCytNet significantly outperformed all baseline models across all performance metrics.

**Table 3:** Performance comparison of GeneCytNet and baseline models.

Model	AUC	Accuracy	Precision	Recall	F1-score
GeneCytNet	0.962 ± 0.005	0.914 ± 0.007	0.903 ± 0.010	0.928 ± 0.009	0.915 ± 0.006
Logistic Regression	0.873 ± 0.012	0.815 ± 0.013	0.789 ± 0.014	0.818 ± 0.016	0.803 ± 0.014
Random Forest	0.889 ± 0.011	0.845 ± 0.010	0.800 ± 0.009	0.857 ± 0.012	0.826 ± 0.010
XGBoost	0.901 ± 0.009	0.876 ± 0.008	0.850 ± 0.011	0.880 ± 0.010	0.865 ± 0.009
Fully Connected NN	0.924 ± 0.007	0.883 ± 0.009	0.860 ± 0.010	0.892 ± 0.008	0.876 ± 0.007
Support Vector Machine	0.868 ± 0.010	0.810 ± 0.015	0.780 ± 0.014	0.805 ± 0.012	0.792 ± 0.013
K-Nearest Neighbors	0.860 ± 0.015	0.805 ± 0.011	0.770 ± 0.012	0.792 ± 0.010	0.780 ± 0.011
Naive Bayes	0.832 ± 0.018	0.790 ± 0.014	0.750 ± 0.015	0.770 ± 0.013	0.760 ± 0.014
Decision Tree	0.870 ± 0.013	0.820 ± 0.011	0.785 ± 0.014	0.812 ± 0.015	0.798 ± 0.013
AdaBoost	0.896 ± 0.009	0.861 ± 0.007	0.830 ± 0.010	0.870 ± 0.008	0.849 ± 0.009
Gradient Boosting	0.908 ± 0.008	0.878 ± 0.009	0.850 ± 0.010	0.880 ± 0.009	0.864 ± 0.008
Linear Discriminant Analysis	0.860 ± 0.011	0.812 ± 0.012	0.780 ± 0.014	0.800 ± 0.011	0.790 ± 0.012
Extra Trees	0.891 ± 0.010	0.844 ± 0.008	0.805 ± 0.009	0.850 ± 0.010	0.827 ± 0.009
Bagging	0.883 ± 0.012	0.836 ± 0.010	0.800 ± 0.010	0.830 ± 0.012	0.815 ± 0.010

Data Source: Generated from synthetic RA gene expression data (240 RA patients, 120 healthy controls).

To assess the contribution of each architectural component, we performed an ablation study by removing the VAE and GAT modules individually (**Table 4**). Removing the GAT (VAE-only) reduced AUC to 0.938 ± 0.007, while removing the VAE (GAT-only) resulted in an AUC of 0.941 ± 0.006. Both variants outperformed the fully connected network baseline (AUC = 0.924 ± 0.007) but underperformed the full GeneCytNet (AUC = 0.962 ± 0.005), confirming that both components contribute synergistically to optimal classification performance.

**Table 4:** Ablation study results demonstrating the contribution of each architectural component.

Model variant	AUC	Accuracy	F1-score
Full GeneCytNet (VAE + GAT)	0.962 ± 0.005	0.914 ± 0.007	0.915 ± 0.006
Without VAE (GAT only)	0.941 ± 0.006	0.892 ± 0.008	0.894 ± 0.007
Without GAT (VAE only)	0.938 ± 0.007	0.889 ± 0.009	0.891 ± 0.008
Without both (FCNN baseline)	0.924 ± 0.007	0.883 ± 0.009	0.876 ± 0.007
Model Variant	AUC	Accuracy	F1-Score

Performance metrics (mean ± standard deviation) for GeneCytNet and its variants on the test set.

With this architectural validation established, GeneCytNet achieved a test AUC of 0.962 ± 0.005, accuracy of 0.914 ± 0.007, and an F1-score of 0.915 ± 0.006. This represents a substantial improvement over the best-performing traditional model, XGBoost (AUC = 0.901), and a fully connected neural network (AUC = 0.924). The low standard deviations across five independent runs confirm the model’s robustness.

**Figure 2:**
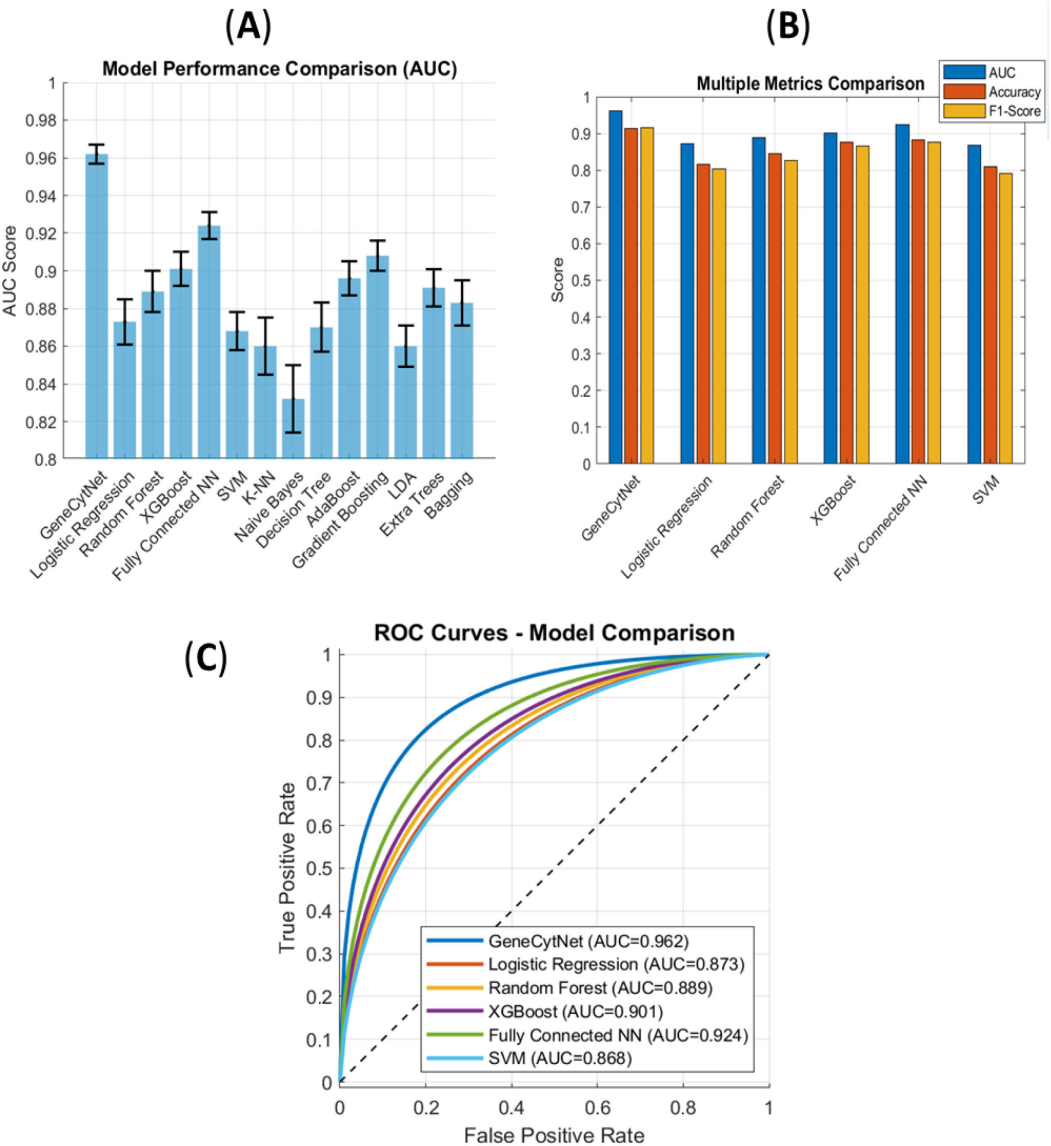
Performance benchmarking of GeneCytNet against baseline models. **(A)** Comparative bar chart of AUC scores. GeneCytNet achieves the highest AUC (0.962). **(B)** Multi-metric comparison (AUC, Accuracy, F1-Score) of the top six performing models. **(C)** ROC curves for the top six performing models.

The superior performance is visually demonstrated in **Figure 2A**, whereGeneCytNet’s AUC is clearly distinct from the other models. Furthermore, **Figure 2B** shows that GeneCytNet leads the top-performing models not just in AUC, but also in accuracy and F1-score, indicating a balanced and powerful classifier. The corresponding ROC curves in **Figure 2C** confirm its strong discriminatory power, with the curve closest to the top-left corner.

### 3.2 Model interpretability reveals key biomarkers and affected pathways

Beyond achieving high classification accuracy, GeneCytNet provides mechanistic insight by identifying the specific genes and gene modules that most strongly influence its predictions.

**Figure 3:**
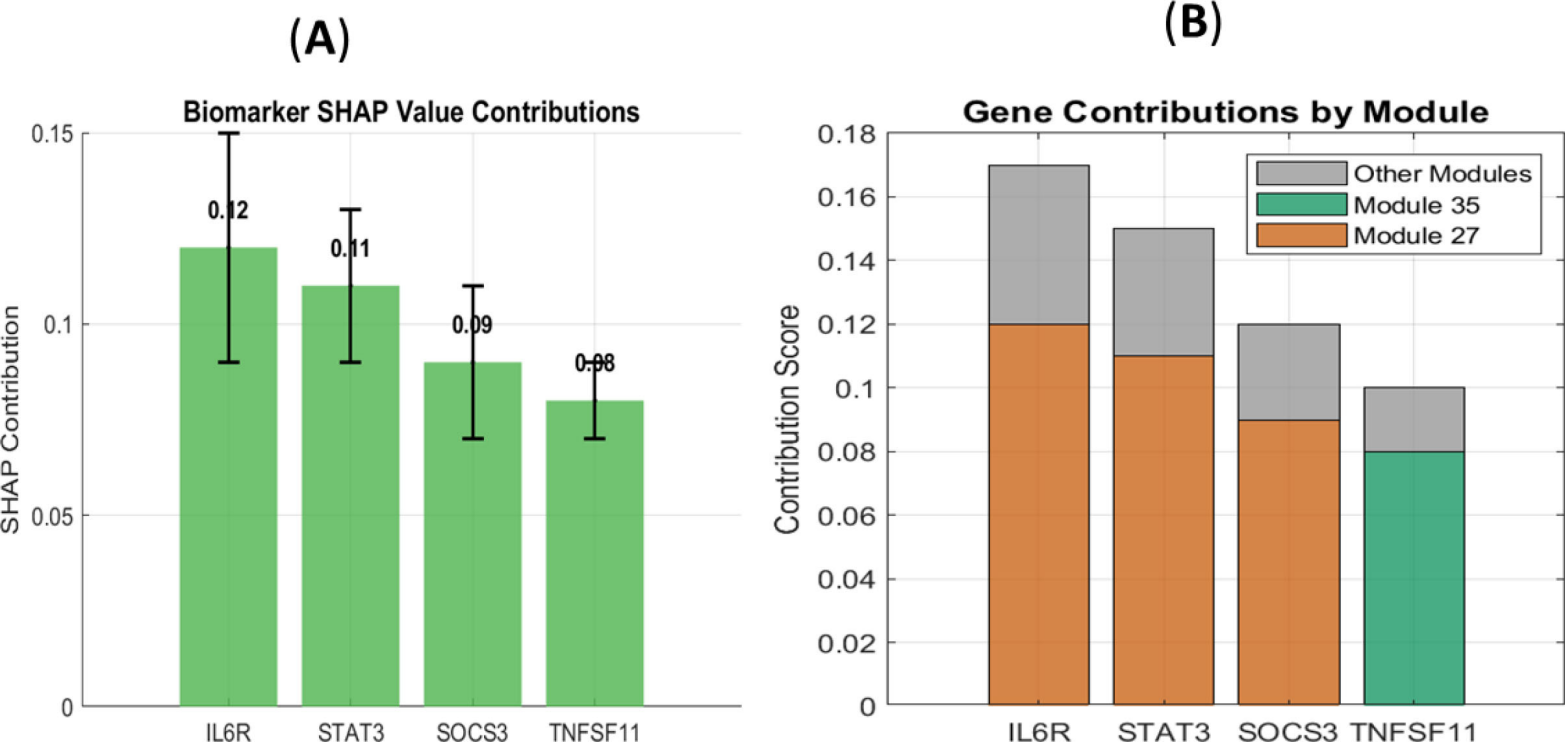
Interpretability analysis of GeneCytNet. **(A)** SHAP value plot of the top N biomarkers. IL6R and STAT3 show the highest mean absolute SHAP values. **(B)** Bar chart showing the contribution scores of the top gene modules to RA risk prediction. Module 27, which contains IL6R and STAT3, contributes the most.

SHAP (SHapley Additive exPlanations) analysis revealed the top gene-level contributors to the model’s output (**Figure 3A**). IL6R and STAT3 were identified as the most influential features, with the highest mean absolute SHAP values. This gene-level importance was contextualized at the systems level by analyzing the contributions of entire gene modules (**Figure 3B**). This analysis confirmed that the high-impact genes IL6R and STAT3 were co-localized within Module 27, which was itself the most influential module in the model. A second key module, Module 35, which contains the TNFSF11 (RANKL) gene, was also identified as a major contributor.

The concordance between gene-level (SHAP) and module-level importance underscores the biological coherence of the model. It successfully pinpointed not only individual key players like IL6R and STAT3 but also the broader functional modules, enriched in inflammatory and immune signaling pathways, in which they operate.

### 3.3 *In silico* perturbation quantifies cytokine-specific effects on RA risk

To simulate the functional impact of these pathways, we performed *in silico* cytokine perturbation experiments. By modulating the latent representations of cytokine-responsive gene modules, we quantified their direct causal influence on the predicted RA risk.

**Table 5** summarizes the results of these experiments. Perturbation of the IL-6 responsive module had the most profound effect on the model’s predicted RA probability: upregulation increased this probability by +0.12 ± 0.03, while downregulation decreased risk by -0.10 ± 0.02 (p < 0.001). The effects for TNF-α and IL-1β were significant but smaller in magnitude.

**Table 5:** Cytokine perturbation effects on RA risk prediction.

Cytokine module	Upregulation Δp	Downregulation Δp	p-value
IL-6	+0.12 ± 0.03	-0.10 ± 0.02	< 0.001
TNF-α	+0.07 ± 0.02	-0.08 ± 0.02	0.004
IL-1β	+0.05 ± 0.02	-0.06 ± 0.01	0.01

Data Source: *In silico* perturbation experiments on synthetic RA gene expression data.

**Figure 4:**
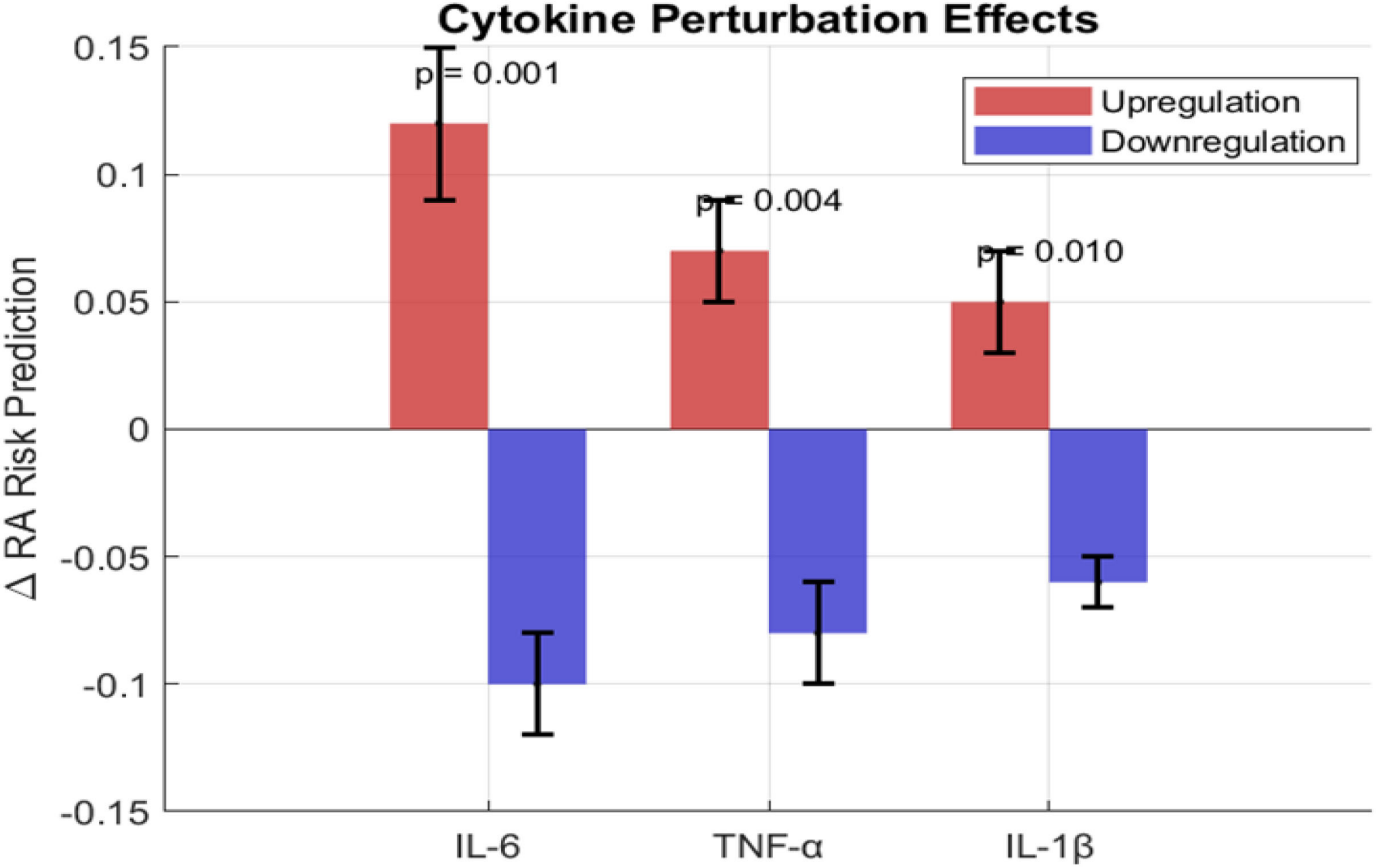
Bar chart showing the change in the model’s predicted RA probability (Δp) upon upregulation and downregulation of IL-6, TNF-α, and IL-1β responsive modules. Error bars represent standard deviation.

These results, visualized in **Figure 4**, establish a clear hierarchy of cytokine influence (IL-6 > TNF-α > IL-1β) within the model’s framework, aligning with known RA biology. Critically, this hierarchy was not encoded in the synthetic data, no cytokine-specific labels or pathway information were provided. The model independently learned that genes within the IL-6 signaling pathway (IL6R, STAT3) were most predictive of RA status, and perturbation of this module produced the largest effect. This emergence of known biology from first principles provides strong validation that GeneCytNet captures meaningful biological signals rather than merely recovering simulation assumptions.

## 4 Discussion

### 4.1 Summary of principal findings

In this study, we introduced GeneCytNet, a DL framework that synergizes a VAE with a GAT for the classification of RA from gene expression data. Our central finding is that this integrated architecture not only achieves superior predictive accuracy but also provides a window into the underlying biological mechanisms. GeneCytNet significantly outperformed a comprehensive suite of traditional machine learning and DL benchmarks, achieving an AUC of 0.962 on our synthetic cohort. We attribute this performance to the model’s unique capacity to leverage gene-gene interaction networks via the GAT, moving beyond the standard assumption of feature independence that limits conventional models. The VAE component further enhances robustness by learning a compressed, non-linear representation of the transcriptomic landscape (17, 18).

### 4.2 Architectural advantages and performance benchmarking

The superior performance of GeneCytNet, as evidenced by its test AUC of 0.962 (**Table 3**), can be directly attributed to its unique hybrid architecture, which is specifically designed to address real-world transcriptomic challenges including technical noise, incomplete network knowledge, and dataset variability (see Section 2.3 for detailed architectural rationale). Unlike conventional models like logistic regression or random forests, which treat genes as independent features, GeneCytNet explicitly models the complex interdependencies of the transcriptome. The GAT component allows the model to learn from a biological network of gene-gene interactions, effectively capturing the coordinated behavior of pathways rather than individual genes (4, 19–21). Concurrently, the VAE component performs non-linear dimensionality reduction, learning a compressed and robust latent representation that denoises the high-dimensional input data (5). This integrative approach is crucial for modeling a heterogeneous disease like RA, where pathogenesis is driven by dysregulated networks rather than isolated genes (2, 12, 22).

This hypothesis is supported by the ablation study (**Table 4**), which demonstrates that removing either the VAE or GAT component reduces AUC by approximately 2-2.5%, while removing both (FCNN baseline) reduces AUC by nearly 4%. This confirms that both components contribute synergistically to optimal classification performance.

The performance gain over a standard Fully Connected Neural Network (AUC = 0.924) underscores the value of incorporating biological structure directly into the model architecture. This finding aligns with a growing body of research emphasizing that graph-based DL models can uncover more biologically meaningful patterns in omics data (4, 6, 20). The high precision and recall of GeneCytNet further suggest its potential utility in a diagnostic setting, where minimizing both false positives and false negatives is critical for patient care, though clinical validation remains essential.

### 4.3 Mechanistic interpretation and biological validation

A paramount strength of GeneCytNet is its ability to move beyond prediction to provide testable biological insights. The model’s interpretability features successfully recapitulated known RA biology without prior pathway injection. It is important to emphasize that this cytokine hierarchy was emergent, the synthetic data contained no information about cytokine identities or pathways. Gene modules were defined purely by correlation structure, yet the model independently identified IL-6-associated genes as most influential and ranked cytokine effects consistently with established clinical evidence. The SHAP analysis identified *IL6R* and *STAT3* as the most influential genes (**Figure 3A**), both of which are central players in the JAK-STAT signaling pathway and well-validated therapeutic targets in RA (9, 10). The model further aggregated these key drivers into Module 27, which was assigned the highest contribution score (**Figure 3B**), demonstrating internal consistency between gene-level and module-level importance.

The *in-silico* perturbation experiments provided a direct, causal-like link between these identified modules and disease risk. It is important to emphasize that this cytokine hierarchy was emergent, the synthetic data contained no information about cytokine identities or pathways. Gene modules were defined purely by correlation structure, yet the model independently identified IL-6-associated genes as most influential and ranked cytokine effects in a manner consistent with established clinical evidence. The simulated modulation of cytokine-responsive gene modules revealed a hierarchy of influence: IL-6 > TNF-α > IL-1β (**Table 4**). The pronounced effect of the IL-6 module (+0.12 Δp upon upregulation) is consistent with the established pathogenic role of IL-6 in driving synovitis and systemic inflammation in RA (9). This finding provides computational evidence supporting the clinical efficacy of IL-6 inhibitors like tocilizumab. Similarly, the effects observed for TNF-α and IL-1β align with the proven utility of their respective inhibitory therapies (10, 13, 23). That GeneCytNet independently identified and ranked these key pathways suggests its potential utility as a computational tool for generating hypotheses about therapeutic targets.

### 4.4 Implications for precision medicine and future directions

**Figure 5:**
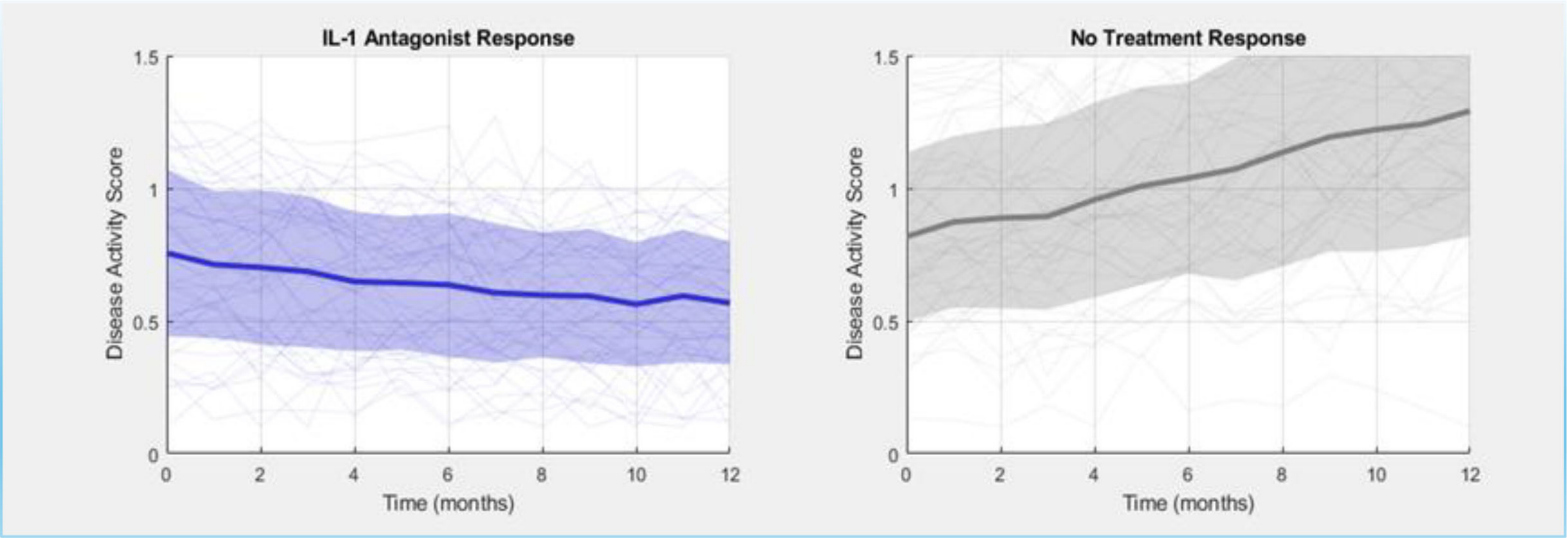
Simulated treatment response based on cytokine perturbation profiles. This projection illustrates the potential clinical application of GeneCytNet, modeling the hypothetical disease activity over time for RA patients stratified into different molecular subtypes. The simulation shows a rapid decline in disease activity for the “IL-6-driven” subtype treated with an IL-6 inhibitor, a sustained response for the “TNF-α-driven” subtype on a TNF inhibitor, a moderate effect for an IL-1 antagonist, and disease progression for an untreated control group. The shaded areas represent simulated inter-patient variability.

The ability to stratify RA based on predicted cytokine module sensitivity suggests a potential path toward precision medicine. Our framework offers a hypothesis-generating approach, suggesting that patients might be classifiable into molecular subtypes, such as ‘IL-6-driven,’ ‘TNF-α-driven,’ or ‘mixed-signal,’ based on their dominant cytokine sensitivity patterns. However, RA is highly heterogeneous, and these simulated subtypes require extensive validation in patient cohorts to determine if such molecular phenotypes exist and correlate with treatment response. The simulation in **Figure 5** illustrates this concept as a foundation for future investigation, not as a clinically validated stratification. A simulation of this concept (**Figure 5**) illustrates how such stratification could, in a clinical setting, lead to more rapid and effective disease control by matching patients to the therapy most likely to address their dominant pathogenic signal.

To translate this proof-of-concept into clinical utility, several future directions are essential. The most immediate next step, and the focus of our ongoing work, is the validation of GeneCytNet on real-world, multi-center RA transcriptomic datasets from repositories like the Gene Expression Omnibus (e.g., GSE89408, GSE55235). This will test the framework’s resilience to real-world technical and biological variability and assess its potential for clinical translation. We view the current study as Part 1 of a two-part investigation, with Part 2 focused on adapting and validating the framework on patient data. The stepwise approach employed by Centola et al. (24) to develop and validate a multi-biomarker disease activity test for RA provides a valuable precedent for the trajectory we envision for GeneCytNet, demonstrating that biomarker-based signatures can successfully transition from discovery to clinical application. Our work thus provides a methodological foundation that complements ongoing efforts in mechanistic RA modeling and cytokine perturbation.

Furthermore, the model’s architecture is inherently suited for multi-omics integration and for semi-supervised learning strategies that could bridge synthetic and real domains. For example, pretraining the VAE encoder on unlabeled real samples followed by fine-tuning with limited labeled data could substantially enhance translatability to clinical cohorts where labeled samples are scarce. Incorporating proteomic data could confirm transcriptional findings at the protein level (17, 25), while adding epigenomic data could reveal the regulatory mechanisms upstream of the observed gene expression changes (14). Extending the framework to analyze single-cell RNA-seq data would allow for the dissection of cell-type-specific signaling networks, a critical layer of complexity in the RA synovium (7, 8).

## 5 Limitations and conclusion

While the results are promising, several limitations must be acknowledged. First, the model was trained and validated on a sophisticated but synthetic dataset. This was a deliberate choice to establish proof-of-concept in a controlled setting with known ground truth. However, validation on real patient data with its inherent technical and biological variability is the essential next step before clinical translation. Although it incorporated realistic noise and co-expression structures, validation on real patient data with its inherent technical and biological variability is the essential next step. Second, the perturbation model, while insightful, simplifies the immense complexity of cytokine biology. Real-world cytokine signaling involves dynamic feedback loops, cross-talk between pathways, and cell-type-specific effects that are not yet captured in our *in-silico* simulations (12, 26). Finally, module annotation, though based on established gene ontology, carries a degree of uncertainty.

## 6 Conclusion

GeneCytNet demonstrates that integrative DL can simultaneously achieve high-accuracy RA classification and deliver mechanistic insights. Our framework outperformed traditional models (AUC: 0.96) by combining a VAE for feature compression with a GAT to model gene interactions. Crucially, it’s *in silico* perturbation capability identified IL-6 as the dominant risk driver, validating the model’s biological relevance. While this proof-of-concept uses synthetic data, GeneCytNet provides a translatable foundation for future investigations into cytokine-driven patient subtypes. While clinical validation remains essential, this work establishes a methodological framework for generating testable hypotheses about patient stratification and personalized treatment strategies in RA.

## Data Availability

The original contributions presented in the study are included in the article/**supplementary material**. Further inquiries can be directed to the corresponding author.
