# Supplementary material for: GeneCytNet: an interpretable deep learning framework for rheumatoid arthritis classification and in silico cytokine perturbation modeling
Source: Front Immunol. 2026 Mar 17;17:1738625. doi: 10.3389/fimmu.2026.1738625 (PMC13036109; doi:10.3389/fimmu.2026.1738625)
Supplement: Supplementary file 1 [file Table1.docx]

**GeneCytNet: An Interpretable Deep Learning Framework for Rheumatoid Arthritis Classification and *In Silico* Cytokine Perturbation Modeling**

**Authors:** Chen Chen**^1^**^*^, Dagang Li**^1^**, Lujia Xu**^2^**

**Address:**

**^1^** Department of Orthopedics, the First Affiliated Hospital of Xiamen University, School of Medicine, Xiamen University, Xiamen 361000, Fujian, China.

**^2^** Xiamen University Tan Kah Kee College, Xiamen province, Zhangzhou city, 363123, China.

**Correspondence:** * Chen Chen**^1^**, **E-mail:**  chen-chen2@ldy.edu.rs

**Co-Authors:** Dagang Li**^1^**, **E-mail:** lidagang@sina.com

Lujia Xu**^2^**, E-mail: 770372134@qq.com

**Supplementary data**

**Table S1.** Sensitivity analysis of gene co-expression graph construction.

| **Correlation Threshold** | **Number of Edges in Graph** | **Test AUC** | **Test Accuracy** | **Test F1-Score** |
| --- | --- | --- | --- | --- |
| Top 0.5% | ~560,000 | 0.958 ± 0.006 | 0.909 ± 0.008 | 0.910 ± 0.007 |
| **Top 1%** | **~1,125,000** | **0.962 ± 0.005** | **0.914 ± 0.007** | **0.915 ± 0.006** |
| Top 1.5% | ~1,690,000 | 0.959 ± 0.005 | 0.911 ± 0.008 | 0.912 ± 0.007 |
| Top 2% | ~2,250,000 | 0.955 ± 0.006 | 0.907 ± 0.009 | 0.908 ± 0.008 |
| Top 5% | ~5,620,000 | 0.941 ± 0.008 | 0.895 ± 0.011 | 0.897 ± 0.010 |

Performance of GeneCytNet across different correlation thresholds for edge inclusion in the graph. Metrics are reported as mean ± standard deviation from five independent runs. The top 1% threshold was selected for the final model.


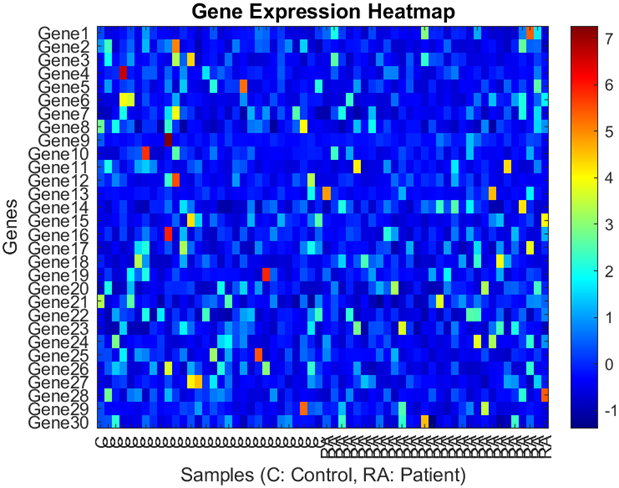


**Figure S1.** Heatmap of top 30 differentially expressed genes. Shows expression patterns in control vs. RA groups from the synthetic dataset. Red indicates high expression; blue indicates low expression.
